# Optimising Combat Readiness: Practical Strategies for Integrating Physiological and Psychological Resilience in Soldier Training

**DOI:** 10.3390/healthcare12121160

**Published:** 2024-06-07

**Authors:** José Francisco Tornero-Aguilera, Maria Stergiou, Alejandro Rubio-Zarapuz, Alexandra Martín-Rodríguez, Luís Miguel Massuça, Vicente Javier Clemente-Suárez

**Affiliations:** 1 Faculty of Sports Sciences, Universidad Europea de Madrid, Tajo Street, s/n, 28670 Madrid, Spain; josefrancisco.tornero@universidadeuropea.es (J.F.T.-A.); stergioumaria89@gmail.com (M.S.); alejandro.rubio@universidadeuropea.es (A.R.-Z.); sandra.martin.rodriguez8@gmail.com (A.M.-R.); vicentejavier.clemente@universidadeuropea.es (V.J.C.-S.); 2 ICPOL—Police Research Center, Higher Institute of Police Sciences and Internal Security, 1300-663 Lisbon, Portugal; 3 Centro de Investigação em Desporto, Educação Física, Exercício e Saúde (CIDEFES), Lusófona University, 1749-024 Lisbon, Portugal; 4 Centre of Research, Education, Innovation and Intervention in Sport (CIFI2D), Faculty of Sport, University of Porto, 4200-450 Oporto, Portugal; 5 Grupo de Investigación en Cultura, Educación y Sociedad, Universidad de la Costa, Barranquilla 080002, Colombia

**Keywords:** psychophysiological interactions, military training, combat stress, psychological resilience, advanced training technologies

## Abstract

This narrative review examines the intricate psychophysiological interplay between cognitive functions and physical responses within military personnel engaged in combat. It elucidates the spectrum of responses elicited by symmetric and asymmetric warfare alongside specialised combat scenarios, including close-quarters and subterranean warfare. Central to this discourse is the emphasis on integrating training programs beyond physical conditioning to encompass psychological resilience and decision-making efficacy under duress. The exploration further ventures into applying advanced technologies such as virtual reality and wearable devices, highlighting their pivotal role in augmenting training outcomes and supporting soldier health. Through a detailed analysis of psychophysiological variations across different military branches of service, the narrative review advocates for bespoke training regimens and support frameworks tailored to address the unique exigencies of each service branch. Concluding observations stress the importance of evolving military training paradigms, advocating for adopting realistic, immersive training simulations that mirror the complexities of the contemporary battlefield. This synthesis aims to contribute to the ongoing discourse on optimising military training protocols and enhancing the operational readiness and well-being of armed forces personnel. This narrative review is essential for military psychologists, trainers, and policymakers, aiming to bridge the gap between theoretical knowledge and practical implementation in military training programs.

## 1. Combat Mind and Body: The Soldier’s Psychophysiological Response

The convergence of mind and body within the dynamic realm of military combat constitutes a nuanced and pivotal focus in military psychology and operational effectiveness [1]. Amidst the rigours of combat scenarios, soldiers manifest a spectrum of psychophysiological responses, intricately shaping their decision-making, performance, and overall well-being [2]. These profound reactions, ignited by the formidable demands of warfare, stand as a testament to the intricate interplay between psychological processes and physiological responses under the relentless strain of stress [3]. Examining this intricate intersection unveils the intricacy between the cognitive and physiological aspects, shedding light on the profound impact this relationship has on the adaptability and resilience of military personnel in high-stakes situations. Central to understanding these responses lies the orchestrated activation of the sympathetic nervous system, a pivotal element in the body’s acute stress response [4]. This intricate ‘fight or flight’ mechanism orchestrates a symphony of immediate physiological transformations, including heightened heart rate, accelerated breathing, and intensified alertness, designed to prepare the body for potential threats. In the crucible of combat, these responses become indispensable, empowering soldiers to swiftly and effectively counter immediate dangers [5,6]. The essential role of these reactions in a military context accentuates the imperative need for meticulously crafted training and support systems. These systems should be finely tuned to bolster soldiers’ resilience in the relentless face of combat stress, recognising the critical interplay between the physiological mechanisms at play and the psychological fortitude required for optimal performance and well-being on the battlefield [7]. However, the psychophysiological ramifications of combat transcend the immediacy of initial responses. Extended exposure to the rigours of combat stress can give rise to an array of enduring psychological and physiological challenges [8]. Of notable concern is the emergence of chronic stress, marked by heightened cortisol levels, a factor with the potential to detrimentally impact cognitive functions and decision-making abilities over the long term [9]. On the physiological front, sustained stress can induce adverse consequences, including fatigue and compromised immune function [4]. Concurrently, the psychological toll becomes evident, correlating with an elevated risk of debilitating conditions such as anxiety, depression, and post-traumatic stress disorder (PTSD) [9]. This underscores the imperative for comprehensive and sustained support mechanisms, acknowledging the enduring repercussions that extended combat exposure can have on the mind and body of military personnel.

Furthermore, the cognitive and emotional dimensions of a soldier’s response in combat are equally paramount to the physical facets. The intense stress milieu of combat fosters heightened vigilance and alertness, a double-edged sword that, while advantageous in refining reaction times, can precipitate cognitive overload and subsequent lapses in judgment. In this intricate balancing act, emotional regulation emerges as a pivotal skill, demanding that soldiers adeptly navigate a spectrum of emotions ranging from fear to exhilaration [10]. Each emotional nuance possesses the potential to influence their behaviour significantly and the intricacies of their decision-making processes, highlighting the need for soldiers to master the art of emotional resilience amidst the tumultuous landscape of combat [11]. Given the profound implications of these psychophysiological responses in combat, a comprehensive understanding and strategic addressing of these phenomena are imperative prerequisites for evolving efficacious military training programs [12]. These initiatives must transcend the conventional emphasis on physical preparedness alone, extending their purview to cultivating essential skills enabling soldiers to adeptly manage stress, regulate emotions, and uphold cognitive clarity amidst pressure. The overarching goal is cultivating a military force that transcends physical prowess, embodying psychological resilience to withstand combat’s relentless challenges while sustaining optimal performance levels [1]. This holistic approach not only fortifies soldiers for the physical demands of warfare but also equips them with the mental fortitude essential for navigating the complexities of the battlefield with unwavering efficacy [1].

### Methodology

In recognition of the profound implications arising from soldiers’ psychophysiological responses in combat, the primary aim of this narrative review is to thoroughly examine the current understanding of these responses and their far-reaching impact on military training and overall well-being. We aim to unveil the intricate pathways and mechanisms through which soldiers’ mental and physical health are influenced across diverse combat environments. We propose innovative military training approaches to bolster resilience and elevate performance. To achieve our objective, we explored the primary and secondary literature, encompassing scientific journals, bibliographic repositories, and platforms such as PubMed, Scopus, Embase, Science Direct, Sports Discuss, ResearchGate, and the Web of Science. Our search leveraged terms compatible with military psychology and physiology, including combat stress, PTSD, soldier performance, resilience training, military nutrition, tactical training, psychophysiological responses, and stress management in soldiers. Our narrative review spanned articles published from 1 September 2003 to 1 September 2023. We set specific exclusion parameters: (i) works not aligned with the central theme of soldiers’ responses in combat; (ii) doctoral theses, symposium summaries, and non-published materials. To ensure the thoroughness and relevance of our review, we initially identified a total of 450 articles through our extensive search across multiple databases. After applying our specific exclusion criteria and rigorous appraisal, we retained 120 articles that were directly pertinent to our research objectives. Then, after careful revision, 90 articles were included. This selection process underscores our commitment to providing a comprehensive and precise analysis of the psychophysiological responses of soldiers in diverse combat scenarios. A panel of six reviewers diligently appraised the titles and summaries of all gathered documents to determine their relevance. Manuscripts utilising outdated information, bearing topics not congruent with our research goals, or not written in English were discarded. The same reviewers responsible for the paper selection independently culled essential information from the chosen articles. The utilisation of artificial intelligence tools was implemented during the figure creation process to enhance their visual appeal and increase their clarity for the reader. This utility has been bing.com, a Microsoft-owned and operated search engine. Furthermore, AI-based translation tools were used as a supplementary measure to refine language. We uphold the highest standards of academic integrity and transparency, and the scientific content of our work (core research, analysis, interpretation, and writing) is conducted by the authors without the aid of AI and is entirely original and human-authored.

## 2. Brain and Body on the Battlefield: Decoding Combat Types and Responses

When subjected to the rigours of diverse combat scenarios, the intricate interplay between the brain and body is a focal point of rigorous investigation within military science [13,14]. Researchers diligently strive to unravel the complex web of physiological and psychological responses evoked by distinct combat environments, aiming to decode their nuanced implications for soldier performance and overall well-being. 

### 2.1. Symmetric Combat

Symmetric combat unfolds in confrontations where opposing forces boast comparable military capabilities and resources, setting the stage for battles where strategic and tactical acumen becomes paramount in determining outcomes [15]. The parity in strength presents distinctive psychological and physiological challenges for soldiers, amplifying the complexity of their experiences on the battlefield. Furthermore, the equilibrium in capabilities has profound implications for military strategy and training, necessitating a nuanced approach considering the intricacies inherent in engagements where forces are evenly matched [16].

Symmetric combat, delineated by the meticulous alignment of technological, tactical, and personnel strengths, paints a canvas where adversaries engage in an intricate dance of balance and equivalence. This equilibrium sets the stage for a high-intensity conflict environment. This dynamic arena places formidable demands on soldiers, requiring not only skilful execution of tactics but also a profound level of adaptability and resilience. Within the crucible of symmetric combat, the psychological impact on soldiers is profound and intricate [17]. The meticulous balance of forces elevates stress levels among soldiers, a consequence intricately woven into the unpredictability and intensity inherent in confrontations with an equally matched adversary. This heightened stress response becomes a palpable force, propelling soldiers toward increased vigilance—an essential attribute in navigating the complexities of symmetric conflicts [18]. However, this heightened state of stress also ushers in a higher susceptibility to combat-related anxiety, underscoring the multifaceted nature of the challenges soldiers confront in such scenarios. Unravelling the layers of symmetric combat reveals a terrain marked by strategic intricacies and where soldiers become psychological warriors, navigating the fine line between heightened vigilance and the potential toll on mental well-being [19]. Recognising and addressing these psychological dimensions becomes integral in fortifying soldiers for the unique challenges of symmetric conflicts, ensuring their tactical proficiency and mental resilience in the face of intense and evenly matched encounters. From a physiological standpoint, soldiers engaged in symmetric combat scenarios exhibit reactions reminiscent of those observed in high-intensity athletic competitions. Elevated adrenaline and cortisol levels are noteworthy indicators of the acute stress response [20]. While this physiological reaction can amplify performance in the immediate term, it also carries the potential for enduring health implications if not carefully and comprehensively managed. The intricate interplay between the heightened stress response and its impact on the body necessitates nuanced strategies to mitigate any potential long-term health risks associated with the demands of symmetric combat scenarios [21].

Moreover, regarding combat strategy, symmetric engagements demand a distinctive approach compared to asymmetric warfare. Within symmetric combat, the spotlight turns to conventional warfare tactics, where manoeuvrability and firepower assume central roles. This shift underscores the imperative for soldiers to undergo rigorous training in traditional combat skills [22]. Additionally, it places a premium on cultivating attributes such as mental toughness and strategic thinking, recognising the multifaceted demands and complexities inherent in symmetric combat scenarios. Embracing these aspects becomes crucial for ensuring operational effectiveness and success in the dynamic landscape of evenly-matched conflicts [23].

Further on, the intricacies of symmetric combat wield a profound impact on military training and preparedness. They are emphasising the need for military training programs to replicate the conditions of symmetric warfare. Advanced simulation technologies play a pivotal role in this emulation, effectively exposing soldiers to the multifaceted mental and physical stimuli they are likely to encounter. Such sentiments resonate across various researchers who advocate for integrating virtual reality and other immersive training methods as transformative tools in enhancing soldiers’ readiness for the nuanced demands of symmetric combat scenarios [24]. Additionally, symmetric combat exerts profound implications on military leadership and decision-making. Leadership in symmetric engagements demands a delicate equilibrium between tactical acumen and the ability to sustain morale amid highly stressful conditions. Success in such evenly-matched situations hinges upon effective communication and adept decision-making, illuminating leadership’s pivotal role in navigating the complexities of symmetric combat scenarios [25]. This dynamic underscores the need for leaders to possess strategic insight and foster a resilient and cohesive unit capable of navigating the challenges of symmetric warfare.

Furthermore, military technology in symmetric combat catalyses innovation and progress. Empirical evidence highlights that symmetric conflicts frequently trigger swift advancements in military technology, driven by the competitive endeavours of each side to secure a strategic advantage within the parameters of their comparable capabilities. This dynamic competition fosters an environment where cutting-edge developments and breakthroughs emerge, propelling the evolution of military technology to new heights [26]. Finally, the well-being and psychological health of soldiers in symmetric combat is of paramount concern. Scholarly research consistently emphasises the crucial role of psychological support and counselling for soldiers immersed in these intense and high-stress environments. Such proactive measures prove instrumental in mitigating the potential risks associated with long-term mental health issues, demonstrating a steadfast commitment to safeguarding the overall mental resilience of military personnel engaged in symmetric combat scenarios [1].

### 2.2. Asymetric Combat

Asymmetric combat represents a form of warfare characterised by profound disparities between opposing forces regarding military strength, resources, or tactics. This imbalance creates distinctive challenges across psychological, physiological, and strategic dimensions, necessitating specialised military training and operational planning approaches. The intricate nature of asymmetry demands nuanced strategies and training methodologies to effectively navigate the complexities posed by the parties’ varying strengths, resources, and tactics [27].

In the dynamics of asymmetric combat, one side typically commands a substantial advantage in technology, weaponry, or numerical strength, juxtaposed with the opposing side’s reliance on unconventional tactics. Scholars categorise asymmetric warfare as engagements wherein smaller, less-equipped forces confront more potent adversaries, deploying guerrilla tactics, ambushes, and improvisation. These dynamics introduce distinctive stressors for soldiers, subjecting them to the challenges of navigating unpredictable and unconventional threats in the field [28]. The psychological repercussions of engaging in asymmetric combat are profound. Soldiers immersed in irregular warfare contend with heightened uncertainty and fear stemming from the unpredictable nature of their adversaries’ tactics. This unpredictability induces a perpetual state of heightened alertness, which, over time, can culminate in chronic stress and anxiety [6,29]. The enduring exposure to such unpredictable circumstances exacts a toll on the mental resilience of soldiers, underscoring the imperative for comprehensive psychological support and coping mechanisms to mitigate the long-term effects of these stressors.

Moreover, from a physiological perspective, soldiers involved in asymmetric warfare exhibit distinct stress responses when contrasted with those engaged in symmetric combat. The irregular and sporadic nature of asymmetric engagements gives rise to a fluctuating pattern of stress hormones, which can impact long-term health and cognitive function. The unique and unpredictable stressors inherent in asymmetric warfare underscore the importance of a nuanced understanding of these physiological responses, emphasising the need for tailored health and cognitive support mechanisms for military personnel navigating such challenging environments [1].

Furthermore, asymmetric warfare necessitates a distinct approach to crafting combat strategy and training regimens. Military units embroiled in asymmetric combat must prioritise flexibility, rapid decision-making, and adaptability to navigate unpredictable scenarios effectively. This mandates training programs that emphasise cultivating situational awareness, mastering counter-insurgency tactics, and honing survival skills. The dynamic and unpredictable nature of asymmetric warfare underscores the critical need for military forces to possess a versatile skill set, ensuring their readiness to confront and overcome the diverse challenges inherent in such asymmetrical conflict scenarios. On this line, the role of technology in asymmetric combat is pivotal. Advanced surveillance and reconnaissance technologies emerge as critical components, significantly influencing asymmetric warfare [27]. These cutting-edge tools empower military forces to understand the intricacies of irregular tactics, facilitating more informed and effective countermeasures. Integrating advanced technologies enhances the military’s situational awareness and amplifies its capability to counter adversaries’ unconventional strategies in asymmetric combat scenarios [24]. In addition, leadership challenges in asymmetric warfare are uniquely demanding. Those at the helm in asymmetric combat scenarios must demonstrate exceptional skill in managing uncertainty, nurturing resilience among their troops, and making swift decisions based on limited information [30]. The dynamic nature of asymmetric warfare demands leaders who can navigate ambiguity with precision, instil confidence and adaptability within their teams and demonstrate a keen ability to make critical decisions swiftly and effectively despite the inherent challenges posed by incomplete information.

Moreover, providing psychological support for soldiers engaged in asymmetric warfare is paramount. Where it is imperative to implement robust mental health services to address the distinct stressors associated with irregular warfare, mitigating the risk of conditions such as PTSD and other combat-related mental health issues [31]. The emphasis on comprehensive psychological support serves not only to alleviate immediate stressors but also to foster long-term mental resilience, acknowledging the profound impact that asymmetric warfare can have on the well-being of military personnel. Ultimately, asymmetric warfare carries profound implications for international law and ethics. Upholding international norms and rules of engagement in the context of unconventional warfare tactics becomes challenging [32]. This underscores the ongoing necessity for sustained ethical and legal discourse within military strategy, emphasising the imperative to continuously reassess and adapt ethical frameworks in response to the dynamic landscape of asymmetric warfare.

### 2.3. Combat in Specialised Environments: Understanding Various Scenarios and Mixed Situations

Engaging in combat within specialised environments, ranging from close quarters to underground settings and conflict zones like the Middle East, introduces distinctive challenges demanding tailored strategies and adaptations. These arenas encompass body-to-body combat, subterranean warfare, and operations conducted in regions marked by urban and desert warfare complexities. Meeting the demands of these unique settings necessitates a nuanced approach beyond conventional tactics, underscoring the importance of specialised training and strategic considerations to navigate the intricacies of such diverse combat environments.

#### 2.3.1. Close-Quarters Combat

Close-quarters combat (CQC), often called hand-to-hand combat, introduces distinctive challenges and requisites in military engagements, unfolding when soldiers confront the enemy at extremely close range, frequently within arm’s reach. This form of combat demands an elevated level of physical fitness, mastery of advanced combat skills, and substantial mental preparedness, accentuating the need for a holistic approach to training that encompasses both the physical and mental dimensions essential for success in such intimate and demanding combat scenarios [33].

The preparation for CQC unfolds as a multifaceted endeavour, extending beyond mere mastery of martial arts to encompass extensive psychological conditioning. The overarching goal is to equip soldiers to adeptly navigate the intense stress and fear inherent in the proximity to the enemy. CQC demands rapid reflexes, a heightened level of agility, and significant hand–eye coordination, making rigorous physical training an imperative component [34]. This comprehensive approach aims to forge soldiers capable of seamlessly integrating mental resilience and physical prowess in the demanding dynamics of CQC scenarios. On this line, physical responses in CQC situations are characterised by heightened physiological stress reactions. Within the intense proximity of CQC scenarios, soldiers undergo an escalation in heart rate and an adrenaline rush, potentially enhancing immediate performance but also contributing to accelerated fatigue [33,35]. This underscores the critical need for a comprehensive training regimen integrating skill development and rigorous endurance training [35]. 

From a psychological perspective, CQC introduces distinctive mental challenges. The intimate proximity to the enemy in CQC scenarios has the potential to induce heightened psychological stress, expressed through manifestations of anxiety or increased aggression [34]. As a countermeasure, psychological resilience training assumes paramount importance. This training encompasses stress management and mental conditioning, providing soldiers with essential tools to effectively navigate and cope with the psychological rigours inherent in CQC situations, ensuring their continued effectiveness in these unique challenges [1].

Additionally, cognitive demands in CQC scenarios are significant. Soldiers operating in these environments must rapidly make decisions under intense stress, frequently within unpredictable and dynamic settings. Meeting these demands necessitates more than only tactical knowledge; it calls for the ability to assess and adapt to rapidly changing situations. The cognitive prowess required in CQC extends beyond mere tactical expertise, highlighting the crucial need for soldiers to possess adaptive thinking skills and the capacity for quick decision-making under the pressure of high-stakes and rapidly evolving circumstances [1]. Further, mastering CQC and developing skills for these conditions necessitates a blend of martial arts training and immersive combat scenario simulations [36]. The training regimen must encompass diverse martial arts techniques, weapon-disarming skills, and strategies tailored for close-range combat situations. Emphasising the paramount importance of realistic training scenarios, which authentically simulate the unpredictability and intensity of actual combat, becomes a cornerstone. By integrating these elements, soldiers can refine their technical proficiency and cultivate adaptability for success in CQC engagements’ dynamic and unpredictable nature [37]. Lastly, the emotional toll of CQC must be considered. Participating in combat at such intimate proximity frequently exerts a profound impact on soldiers’ mental health. Exposure to CQC scenarios heightens the risk of PTSD and other mental health issues, underscoring the imperative for comprehensive psychological support and counselling for combat personnel. Recognising and addressing the emotional challenges inherent in CQC engagements is crucial for safeguarding the mental well-being of soldiers and mitigating the potential long-term effects associated with intense and close-quarters combat experiences [29].

#### 2.3.2. Close-Quarters Combat Subterranean Warfare

Subterranean warfare, marked by combat in tunnels and underground facilities, introduces a unique array of tactical and psychological challenges that sharply contrast with traditional above-ground combat. This form of warfare necessitates soldiers to acclimate to confined, poorly lit, and frequently disorienting environments [2].

Mastering navigation and combat within these confining spaces is intricate. Emphasis on specialised training underscores the significance of honing skills in navigation, communication, and small-unit tactics to guarantee effectiveness in these distinctive conditions. The unfamiliarity and confinement of subterranean environments heighten the importance of maintaining a high mental and physical preparedness [38]. In addition to tactical challenges, subterranean warfare imposes considerable psychological stress on soldiers. Research reveals that the claustrophobic nature of underground combat contributes to heightened anxiety and stress levels [2]. The absence of natural light and the enclosed spaces intensify feelings of disorientation and claustrophobia, adversely affecting soldiers’ mental health and combat readiness [35,36,37]. Physiologically, soldiers engaged in subterranean warfare face significant challenges. Operating in these environments often induces heightened fatigue because of the physically demanding nature of navigating confined spaces [39].

Moreover, the subpar air quality and elevated humidity in underground settings can contribute to respiratory issues and dehydration, further intensifying the array of physical challenges soldiers must face. In addition, in subterranean warfare, situational awareness becomes paramount. Soldiers must master swiftly assessing their environment and identifying potential threats, as confined spaces often restrict visibility and manoeuvrability. Achieving this level of awareness demands technical training and a high degree of mental agility and adaptability, enabling soldiers to navigate the complexities of underground environments effectively [40].

Furthermore, communication hurdles in subterranean environments pose a noteworthy challenge. Traditional communication systems frequently prove less effective underground, compelling the need for specialised equipment and protocols. These adaptations are crucial to sustaining efficient team coordination and situational awareness in the intricate and confined spaces characteristic of subterranean warfare [41]. Moreover, the impact of underground warfare on team dynamics is profound. They emphasise the significance of teamwork and unit cohesion in these environments, where individual actions can exert immediate and substantial effects on the safety and success of the entire unit. Finally, training for subterranean warfare must be both comprehensive and realistic. Training programs should incorporate simulations replicating underground environments’ physical and psychological conditions. This includes rigorous exercises in confined space navigation, low-light combat, and emergency medical response tailored to subterranean conditions [34].

#### 2.3.3. Operations in the Middle East and Urban Environments

Military operations in the Middle East, especially within urban environments, introduce intricate challenges that set them apart from traditional battlefield scenarios. These operations, marked by urban warfare and counter-insurgency tactics, demand soldiers to navigate and adapt to densely populated urban terrains while confronting an adversary known for employing guerrilla tactics [42].

Exploring the complexities of urban battles in the Middle East, seasoned experts underscore the imperative for soldiers to familiarise themselves with the distinctive demands of urban warfare. Negotiating these environments requires soldiers to traverse densely populated areas, contending with narrow streets, towering buildings, and civilian populations [43]. This urban landscape mandates a tactical approach prioritising agility, precision, and the capacity to swiftly adapt to evolving scenarios. Additionally, the psychological ramifications of such operations are profound. Confronting an adversary utilising guerrilla tactics in urban settings elevates stress levels and demands heightened vigilance. These engagements’ inherent unpredictability and complexity, compounded by the imperative to distinguish between combatants and non-combatants, impose substantial psychological strain on soldiers [6].

Consequently, implementing effective mental resilience training is imperative to adequately prepare troops for the multifaceted challenges they encounter in these scenarios. Further on, intelligence in urban warfare and counter-insurgency operations is pivotal. The significance of precise and timely intelligence cannot be emphasised enough, as it offers crucial insights into the enemy’s manoeuvres, tactics, and intentions. In addition to this, possessing in-depth local knowledge and a nuanced cultural understanding proves indispensable. These elements facilitate effective navigation of the region’s intricate social and political landscape and contribute significantly to comprehending the complexities, often as intricate as the physical terrain itself [33]. Moreover, due to the critical role of advanced surveillance and reconnaissance technologies in urban environments, these cutting-edge tools empower forces to acquire essential information while mitigating exposure to potential risks. Moreover, the seamless integration of uncrewed aerial vehicles (UAVs) and other remote sensing platforms has ushered in a revolutionary era in intelligence gathering within these dynamic settings [29].

Furthermore, the training regimen for urban operations must meticulously address the unique challenges posed by close-quarters combat, building clearance, and urban sniping. Integrating simulations and realistic training exercises that faithfully replicate urban conditions is indispensable, ensuring that soldiers are thoroughly prepared for the intricate realities of urban warfare [44]. Additionally, successful urban operations frequently necessitate collaboration with local forces and populations. Establishing trust and cooperation with local entities emerges as a pivotal factor in the overall success of these operations. This emphasises the imperative of fostering cultural sensitivity and honing practical communication skills among soldiers to navigate the complexities of urban environments [45].

In summary, engagements in the Middle East and urban environments demand a holistic approach, addressing tactical adaptability, psychological resilience, advanced intelligence capabilities, and specialised training. The intricacies of these environments mandate that soldiers be prepared physically, tactically, mentally, and culturally to adeptly navigate the diverse challenges of urban warfare and counter-insurgency operations. As urban landscapes persist as pivotal battlegrounds, comprehending and preparing for these distinctive challenges remains paramount.

Furthermore, desert and mountain warfare, pervasive in regions such as the Middle East, unfolds within unique and formidable terrains that demand specialised adaptations to extreme conditions. These environments necessitate physical resilience and present distinctive physiological and psychological challenges that require strategic consideration for effective military operations.

Desert warfare is marked by extreme environmental conditions featuring high temperatures, minimal shade, and expansive, open terrains. The physiological challenges confronting soldiers in these harsh climates are considerable. A primary concern is dehydration due to the intense heat and limited water sources. Furthermore, the risk of heatstroke significantly escalates in these conditions, underscoring the need for thorough acclimatisation and effective hydration strategies for troops [46]. The psychological challenges in desert warfare are formidable. The isolation and monotonous landscape can contribute to decreased morale and mental fatigue among soldiers. The monotony and isolation of desert environments often pose difficulties, affecting soldiers’ mental resilience and effectiveness. To address these psychological challenges, implementing coping strategies such as maintaining regular communication and fostering a strong sense of unit cohesion becomes essential [47].

Mountain warfare, on the other hand, entails operations in high altitude and rugged terrains, often characterised by cold weather and challenging navigation [48]. The physiological demands of mountain warfare are substantial, requiring soldiers to navigate challenges such as hypoxia due to lower oxygen levels at high altitudes, increased caloric and nutritional needs, and the risk of cold-weather injuries like frostbite and hypothermia [49]. In addition, the psychological aspects of mountain warfare encompass coping with isolation and the stress of navigating treacherous terrain. The unpredictable weather and the physically demanding nature of mountainous terrains can exert significant psychological stress on soldiers. The fear of avalanches, landslides, and the challenges in securing supply lines amplify the mental strain [50]. Both desert and mountain warfare demand specialised training and equipment. Soldiers must undergo training in survival skills tailored to these environments, encompassing navigation, shelter-building, and the ability to recognise signs of weather-induced health issues [45,49].

Moreover, operational effectiveness hinges on providing specialised equipment, including suitable clothing, hydration systems, and mountain gear. Furthermore, providing logistical support in these environments poses a complex challenge. Ensuring a continuous supply of essentials such as food, water, and medical supplies becomes particularly demanding in the unpredictable and inhospitable terrains of deserts and mountains. Implementing innovative logistical solutions and establishing reliable communication systems are indispensable for sustaining operations in these areas [49].

In conclusion, desert and mountain warfare introduce distinct challenges demanding thorough and specialised preparation. Grasping the unique physiological and psychological demands inherent in these environments is pivotal for the successful training and operation of military personnel. Formulating customised strategies and providing suitable equipment and support is imperative to guarantee the safety and effectiveness of soldiers navigating these demanding conditions.

## 3. Varied Battlegrounds: Psychophysiology across Military Branches of Service

**Figure 1:**
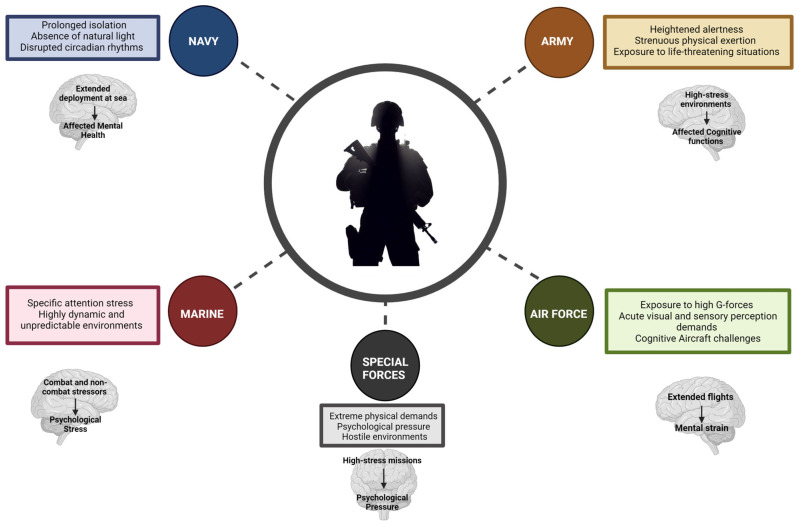
Challenges faced by different soldiers based on their armed forces roles and the mental health issues troops may experience.

Within the intricate landscape of military operations, the psychophysiological responses of personnel exhibit considerable diversity across distinct military branches of service, shaped by the intricacies of their roles, environments, and the specific challenges inherent to each (Figure 1). This variability is not only essential for comprehending the psychological resilience and operational readiness of military personnel but also for crafting training and support systems adept at addressing the unique needs inherent in each division:

- The Army, frequently immersed in ground combat, encounters psychophysiological challenges that set it apart from other branches of service. Extended durations of heightened alertness, strenuous physical exertion, and exposure to life-threatening situations are prevalent. The research underscores the profound impact of high-stress environments on cognitive functions and decision-making abilities. Additionally, the physical demands intrinsic to ground combat, including the carriage of heavy equipment and prolonged marching, exert substantial effects on soldiers’ endurance, cognitive function, and overall health [51];
- In contrast, Air Force personnel, especially pilots, confront a distinct array of psychophysiological stressors. The exposure to high G-forces during flight, the demand for acute visual and sensory perception, and the cognitive challenges inherent in operating complex aircraft systems present unique hurdles. Research has underscored these factors’ impact on pilots’ neuropsychological health. Furthermore, the significance of comprehending the psychophysiological effects of extended flights and the mental strain associated with aerial combat and surveillance missions has been highlighted [52];
- Naval personnel, including those serving on submarines, grapple with distinct psychophysiological stressors tied to prolonged isolation, confinement, and the absence of natural light, disrupting circadian rhythms and potentially affecting mental health. Research has delved into the psychological impact of extended deployment at sea. Furthermore, the submarine service’s unique psychological and physiological challenges, including the effects of prolonged underwater deployment on stress and fatigue levels, have been underscored [53];
- Within the Marine Corps, frequently engaged in amphibious and expeditionary warfare, stressors arise from both naval and ground combat environments. Specific attention has been directed toward the challenges of amphibious operations, emphasising the psychological stress linked to these highly dynamic and unpredictable environments. Additionally, research has delved into the cumulative impact of both combat and non-combat stressors on the mental health of Marine Corps personnel, emphasising the imperative for comprehensive psychological support and resilience training [54];
- In special operations, elite forces such as the Navy SEALs and Army Special Forces undergo rigorous training, priming them for high-risk, high-stress missions. These exceptional units encounter distinct psychophysiological challenges, navigating extreme physical demands and psychological pressure and often engaging in covert operations within hostile environments. The psychological resilience of special operations forces has garnered attention, underscoring the significance of mental toughness and emotional regulation within these units. Additionally, research has illuminated the physiological impact of the intense training regimens and operational stressors on these soldiers, accentuating the necessity for specialised health monitoring and support systems [55].

Therefore, a nuanced comprehension of psychophysiological responses among personnel is imperative in the multifaceted landscape of military branches of service. This insight is a cornerstone for crafting precise strategies to bolster mental health support, fortify resilience through training, and optimise operational effectiveness. The inherent variability in psychophysiological responses across diverse branches of service underscores the intricate nature of military operations, underscoring the essential requirement for tailored approaches. These personalised methodologies are pivotal in elevating the well-being and performance of military personnel, catering to the unique challenges presented in combat and non-combat scenarios.

## 4. Enduring the Unseen Battle: Acute and Chronic Stress and Fatigue in Soldiers

The intricate study of psychological and physiological impacts resulting from acute and chronic stress and fatigue in soldiers has yielded valuable insights into the complexities of military service. Acute stress, frequently encountered in immediate and high-intensity situations, elicits a unique set of psychophysiological responses distinct from chronic stress, which builds up gradually over time due to prolonged exposure to challenging conditions.

Acute stress in soldiers manifests through immediate and intense reactions, triggering the body’s ‘fight or flight’ response, marked by a rapid release of adrenaline and cortisol. This surge of hormones results in heightened heart rate, increased alertness, and a surge in energy levels, preparing soldiers for immediate action. Scientific studies have demonstrated that acute stress positively influences short-term memory and cognitive function, facilitating quick and life-saving decision-making [56]. Nevertheless, this heightened state is not sustainable over the long term, potentially leading to fatigue and cognitive overload if the stressor persists or reaches extreme levels. In stark contrast, chronic stress poses a more insidious threat to the health and well-being of soldiers. This persistent stress stems from prolonged exposure to stressful situations, frequently observed in lengthy military operations [57]. The enduring state of alertness and stress response associated with chronic stress can give rise to a spectrum of psychophysiological issues. The continuous release of stress hormones can disrupt normal bodily functions, resulting in sleep disturbances, impaired immune function, and an elevated risk of cardiovascular diseases. On a psychological level, chronic stress is linked to heightened risks of depression, anxiety, and PTSD, significantly impacting the mental health of soldiers [58].

Further, the intricate interplay between acute and chronic stress in military environments adds complexity to the dynamics. Although critical stress responses can offer short-term benefits by providing the essential physiological and cognitive boosts required in combat situations, the transition to chronic stress poses significant risks. The repetition of acute stressors in a military context may propel soldiers into a state of chronic stress, where they persist in a heightened state of alertness and stress response, even in the absence of immediate threats [59]. This prolonged state has the potential to severely impair cognitive functions over time, diminishing operational effectiveness and escalating the risk of long-term health problems. Therefore, effectively managing both acute and chronic stress is paramount in military settings. Comprehensive training programs and support systems emphasising stress resilience and management play a crucial role [18]. These initiatives are designed to equip soldiers with effective coping strategies for acute stress while actively working to mitigate the transition to chronic stress. Evidence supports the efficacy of cognitive–behavioural techniques, mindfulness, and relaxation training in reducing the impact of chronic stress, thereby enhancing overall well-being [58]. Additionally, fostering a supportive environment and ensuring ready access to mental health resources are vital components in addressing the psychological impacts of chronic stress on soldiers [60].

**Figure 2:**
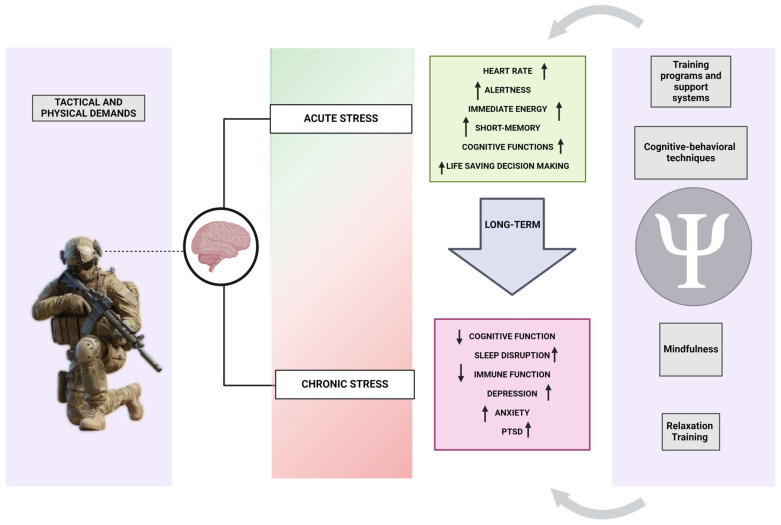
Mental health conditions resulting from continuous acute stress in the military and therapies to enhance it.

In conclusion, a deep understanding of the psychophysiological effects of acute and chronic stress and fatigue is pivotal for promoting military personnel’s well-being and operational effectiveness. Acknowledging that critical stress responses are natural and often beneficial reactions to immediate threats, it is crucial to address the potential risks associated with the transition to chronic stress (Figure 2). Implementing effective management strategies, such as stress resilience training and robust mental health support systems, becomes imperative in navigating these challenges and securing military personnel’s enduring health and effectiveness.

## 5. Mind Tactics: The Art of Psychological Training in the Military

The integration of psychological training in military settings has gained substantial attention, emphasising the crucial synergy between mental fortitude and physical prowess. Psychological training constitutes a diverse array of practices meticulously crafted to elevate cognitive resilience, adept stress management, and the holistic mental well-being of military personnel. A plethora of studies have delved into the efficacy of these interventions, shedding light on their paramount importance in the ever-evolving landscape of modern warfare [61].

A pivotal facet of psychological training is resilience training, meticulously crafted to fortify a soldier’s capacity to endure, rebound, and thrive amidst stressors and challenges [14]. Extensive studies on military veterans have illuminated that resilience training augments adaptability and mental well-being in demanding environments. Furthermore, insights from the Comprehensive Soldier Fitness program underscore the transformative impact of systematic resilience training, showcasing its potential to elevate soldiers’ psychological health and amplify their overall effectiveness. In addition, an integral element of military psychological training is stress inoculation training (SIT) [62]. This method exposes soldiers to controlled stressors in a secure environment, aiming to augment their capacity to cope with stress in authentic scenarios [62]. Research substantiates that SIT significantly diminishes anxiety and enhances performance under stress among military personnel.

Moreover, many studies have firmly established the practical utility of stress inoculation training in effectively preparing individuals for the rigours of high-stress situations [63]. Furthermore, mindfulness and meditation have seamlessly woven into the fabric of psychological training within military contexts. The mindfulness-based Mind Fitness Training (MFT) program has provided compelling evidence that mindfulness training significantly elevates attention and cognitive resilience in high-stress environments.

Furthermore, mindfulness training has demonstrated its dual impact by alleviating stress and augmenting soldiers’ emotional regulation and situational awareness, contributing to a more comprehensive approach to mental well-being [64]. Further on, cognitive–behavioural therapy (CBT) tailored for military applications stands as a formidable tool in confronting specific challenges encountered by soldiers, notably PTSD and anxiety. The application of CBT in the context of PTSD within military personnel has yielded noteworthy outcomes, showcasing substantial reductions in symptoms and associated depression. The effectiveness of CBT in addressing combat-related mental health issues has been emphatically underscored, emphasising its pivotal role in enhancing the psychological well-being of military personnel [65]. Also, the integration of virtual reality (VR) into psychological training signifies a cutting-edge method for readying soldiers to meet the mental challenges of combat. The application of VR in exposure therapy for soldiers with post-traumatic stress disorder (PTSD) has yielded encouraging outcomes, demonstrating its capability to replicate combat scenarios for therapeutic purposes [66] authentically. Beyond its therapeutic applications, VR plays a pivotal role in elevating the authenticity of psychological training, thereby enhancing its overall effectiveness. This innovative approach embraces technological advancements and underscores its potential to reshape and optimise mental preparedness for military personnel [67,68].

In conclusion, the military’s psychological training field is intricate and ever-evolving. Resilience training, stress inoculation, mindfulness, cognitive–behavioural therapy, and virtual reality-based interventions are pivotal components of this multifaceted training. Each methodology provides distinct advantages in equipping military personnel for the intricate psychological demands of military life and combat. 

## 6. Peak of Performance: The Tactical and Hybrid Training and Athlete

The physical preparation of soldiers, often denoted as “military athletes”, is a pivotal facet of military readiness and effectiveness. This preparatory regimen embraces a tactical and hybrid approach to training, seamlessly amalgamating diverse physical conditioning and skill development techniques. The metamorphosis of military training mirrors the evolving exigencies of the battlefield, underscoring a profound acknowledgement of the pivotal role played by physical fitness in the holistic performance of soldiers [69].

Tactical training within the military zeroes in on cultivating the precise physical skills and capabilities essential for combat and military operations. This encompasses a spectrum of attributes, including strength, endurance, agility, and specialised tactical skills like marksmanship, navigation, and hand-to-hand combat. Specialised tactical training programs emerge as catalysts, notably enhancing soldiers’ physical prowess and elevating their combat readiness [70]. The emphasis on bespoke physical training for various military roles resonates in research, underscoring the critical importance of honing specific skills tailored to the demands of diverse military contexts. Nevertheless, hybrid training, a fusion of strength, endurance, and skill components, is gaining increasing recognition as indispensable for readying military personnel to confront the diverse physical challenges that may arise. This comprehensive approach seeks to cultivate a well-rounded physical capability, mitigating the risk of injuries and elevating overall performance [71]. Research consistently affirms the efficacy of hybrid training in enhancing soldiers’ physical fitness, bolstering operational readiness, and fortifying resilience in the face of demanding military scenarios.

Strength and power training are integral beyond merely enhancing muscular strength [72]. Extensive research has illuminated the multifaceted benefits of strength training, extending its positive impacts on bone density, body composition, and injury prevention. Moreover, the significance of power training in military contexts is underscored, emphasising its pivotal role in optimising performance for tasks demanding swift and explosive movements [73]. Moreover, endurance training is a crucial pillar in the comprehensive physical preparation of military personnel. Military operations’ demanding endurance requirements underscore the need for elevated cardiovascular fitness. Robust studies have consistently demonstrated that endurance training enhances soldiers’ aerobic capacity and equips them to execute prolonged physical activities with heightened efficiency [74].

Yet, the role of endurance training is accentuated in fostering soldiers’ resilience, augmenting their ability to sustain high-performance levels over extended periods. In addition, agility and flexibility training constitute integral components in the holistic physical preparation of soldiers. These training modalities enhance soldiers’ adaptability and manoeuvrability in complex and unpredictable environments. Agility training, specifically, sharpens soldiers’ reaction times and refines movement efficiency, proving invaluable in the dynamic scenarios encountered in combat. Simultaneously, flexibility training is a preventive measure, mitigating the risk of musculoskeletal injuries and ensuring soldiers maintain optimal physical functionality [72].

Furthermore, nutrition and recovery are pivotal pillars in the physical preparation of military athletes, encompassing essential components for optimising performance and ensuring long-term readiness. Proper nutrition serves as the bedrock, catering to soldiers’ energy requirements, facilitating efficient recovery, and acting as a preventive measure against injuries. The research underscores the critical role of maintaining adequate energy and nutrient intake, recognising their direct correlation to optimal physical performance [75]. Moreover, the emphasis on recovery extends beyond mere recuperation, playing a crucial role in averting overtraining and fostering sustained physical readiness over the long term [76].

**Figure 3:**
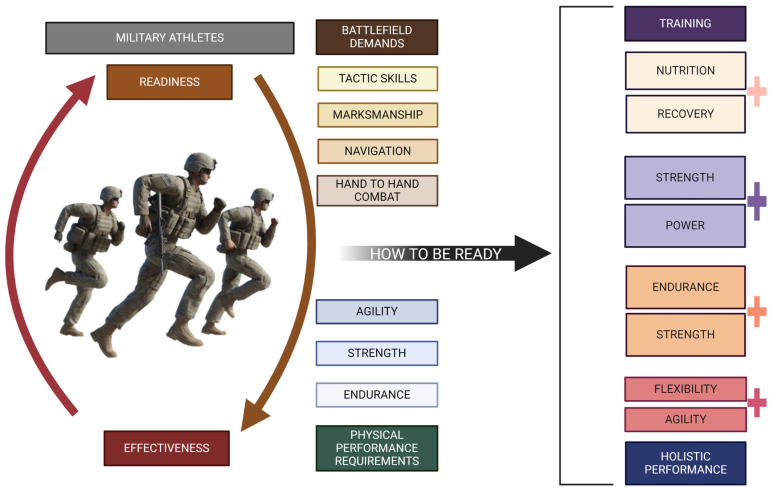
Being successful and prepared for combat requires specialised military training and physical conditioning. Utilising hybrid strength and endurance training, power training, strength training, agility workouts, and flexibility exercises can significantly improve performance. Nutrition and recovery periods will also be critical in this process.

In summary, military athletes’ tactical and hybrid training field represents a dynamic and comprehensive field that adeptly tackles soldiers’ intricate and diverse physical demands. This holistic training approach integrates vital components such as strength, power, endurance, agility, flexibility, nutrition, and recovery, each playing a pivotal role in fortifying soldiers for the demanding rigours of military service (Figure 3). The continuous pursuit of research and development in military physical training is paramount, serving as a linchpin for elevating the performance, resilience, and overall well-being of military personnel, ensuring their preparedness for the multifaceted challenges of modern warfare.

## 7. Tech-Enhanced Warfare: Revolutionizing Military Training and Well-Being

The seamless integration of cutting-edge technology into military training and the holistic management of soldier well-being represents a ground-breaking advancement in modern warfare. This paradigm shift encompasses a spectrum of innovations, ranging from immersive VR and augmented reality (AR) applications in training to the deployment of wearable technology for real-time health monitoring and telemedicine for comprehensive psychological support. These transformative technologies are reshaping the landscape of soldier preparation, combat engagement, and post-mission recovery, ushering in a new era of military effectiveness and resilience [24].

**Figure 4:**
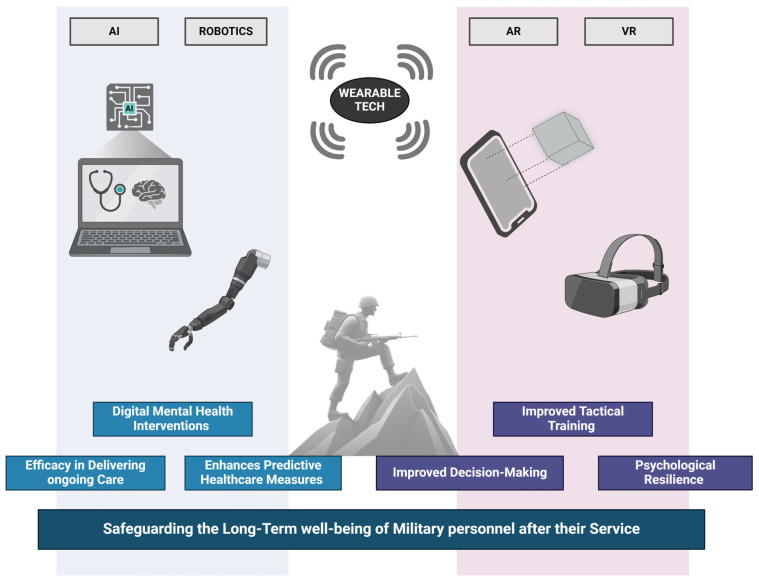
The use of wearable technology helps improve soldiers’ health during military service and beyond. AI (Artificial Intelligence); AR (Augmented Reality); VR (Virtual Reality).

VR and AR integration has proven invaluable in pre-deployment training, offering immersive and lifelike training environments. The effectiveness of VR in elevating combat preparedness is underscored by its ability to simulate scenarios, resulting in improved decision-making and heightened situational awareness among soldiers (Figure 4) [77]. Additionally, AR’s significant impact on navigation and tactical training further accentuates its pivotal role in enhancing practical, real-world operational skills [78]. Furthermore, wearable technology is critical for overseeing soldiers’ physical health and operational readiness during deployment. These devices furnish real-time data on vital signs and stress levels, empowering proactive health and fatigue management. The advantages of wearable tech in operational settings underscore its potential to optimise overall soldier performance [79]. Continuous health monitoring significantly diminishes the risk of injuries and enhances recovery times, contributing to military personnel’s overall well-being and effectiveness [80].

Moreover, digital communication platforms and telemedicine have become indispensable for psychological support during deployment, providing remote access to vital mental health services instrumental in sustaining psychological resilience. The effectiveness of these digital mental health interventions is underscored by substantial improvements in mental health outcomes for deployed soldiers [81]. This technological integration not only enhances accessibility but also plays a pivotal role in nurturing the psychological well-being of military personnel stationed in diverse and challenging environments.

Furthermore, in the post-deployment phase, technology persists as a valuable ally in facilitating the recovery and well-being of soldiers. Telehealth services have demonstrated notable efficacy in delivering ongoing care, especially in remote areas, ensuring that soldiers receive the necessary support regardless of their geographic location [82]. Furthermore, the burgeoning role of robotic rehabilitation technologies in physical recovery is increasingly apparent, with research showcasing their effectiveness in personalised rehabilitation programs. This technological integration not only enhances accessibility to healthcare resources but also contributes significantly to the tailored and effective recovery of military personnel transitioning from deployment [83]. In addition, using data analytics and machine learning in post-deployment health management is a notable advancement. These cutting-edge technologies can predict and proactively address long-term health issues from service-related injuries and stress. Moreover, the application of artificial intelligence (AI) in mental health screenings represents an efficient and forward-thinking approach to identifying and addressing the unique mental health needs of veterans [84]. This integration of advanced technologies enhances predictive healthcare measures and underscores a proactive stance in safeguarding the long-term well-being of military personnel after their service.

In conclusion, the integration of cutting-edge technologies into military training and well-being management signifies a paradigmatic shift in the preparation and care of soldiers. These technologies provide advanced capabilities in training, health monitoring, and recovery and fundamentally enhance the overall effectiveness and well-being of military personnel. As these technologies persistently evolve, they promise to revolutionise the landscape of military training and healthcare, ensuring heightened preparedness and robust support for soldiers throughout all phases of their service (Figure 4).

## 8. From Drill to Field: Real-World Military Training Applications

The evolution of military training from conventional drills to cutting-edge applications that closely replicate real-world scenarios is a pivotal advancement in readying soldiers for the intricacies of modern warfare. This transformation recognises the need for training beyond physical preparedness and tactical skills, prioritising realistic, immersive, and multifaceted training programs. These applications are meticulously crafted to narrow the divide between training environments and authentic combat scenarios, ensuring that soldiers are prepared to confront the diverse challenges they might encounter in the field [85].

In contemporary military training, a ground-breaking application uses advanced simulation systems, frequently incorporating VR and AR. These innovative systems immerse soldiers in environments that faithfully replicate combat scenarios [78]. The inherent advantage of these simulations lies in their capacity to expose soldiers to a diverse array of combat situations within a controlled, repeatable, and secure environment. For example, VR simulations can authentically recreate specific geographical terrains and combat scenarios, enabling soldiers to hone their navigation, decision-making, and tactical manoeuvres pertinent to their designated deployment regions. Rigorous studies have consistently demonstrated that VR-based training significantly enhances spatial awareness, reaction times, and decision-making skills, particularly under stress [77]. This immersive and technology-driven approach fundamentally transforms how soldiers acquire and refine the critical skills necessary for modern warfare.

Moreover, a highly effective application in military training involves the integration of live, virtual, and constructive (LVC) environments. To construct intricate and holistic training scenarios, LVC training seamlessly merges real-world exercises (live), virtual simulations, and computer-generated forces (constructive). This innovative approach offers soldiers an immersive experience that mirrors the unpredictability of real-world combat [86]. It exposes them to scenarios involving interactions with civilians, managing unforeseen events, and making rapid decisions in dynamic and ever-changing situations. The implementation of LVC training has consistently demonstrated its ability to enhance soldiers’ situational awareness and adaptability, which are pivotal skills for success in modern combat operations [86]. This multifaceted and technology-driven training methodology ensures that soldiers are well-prepared for the complexities and challenges they may encounter. In addition, integrating physical and psychological resilience training is paramount in real-world military applications. Specialised programs that emphasise physical endurance, strength, and agility are designed to equip soldiers with the physical preparedness necessary for the demands of combat [87]. Concurrently, psychological resilience training, encompassing stress inoculation and mental toughness exercises, is implemented to fortify soldiers against the unique psychological stresses inherent in warfare. This dual-pronged approach ensures that soldiers are physically resilient and mentally adept in navigating the challenges they will inevitably confront. Extensive research underscores the efficacy of comprehensive resilience training, with documented benefits including enhanced performance, reduced injury rates, and improved mental health outcomes among military personnel [1]. This holistic training strategy is a cornerstone in optimising soldiers’ readiness and overall well-being in the dynamic and demanding contexts of military service.

Furthermore, incorporating wearable technology and biometrics in real-world training scenarios is pivotal in modern military preparation. By continuously monitoring soldiers’ physiological responses during training exercises, instructors gain real-time insights, enabling dynamic adjustments to scenarios that closely mirror the stress and physical demands of actual combat. Wearable tech, offering comprehensive data on heart rate, stress levels, and fatigue metrics, facilitates a nuanced and tailored training approach. This approach maximises training effectiveness by aligning it with individualised physiological responses, ultimately minimising the risk of injury. Robust studies provide compelling evidence for the efficacy of wearable technology, showcasing its capacity to optimise training regimes and enhance overall soldier readiness in the complex and evolving landscape of military operations [79].

Furthermore, incorporating cultural and linguistic training is imperative, particularly for military operations conducted in diverse global environments. Since soldiers frequently engage with local populations, possessing a nuanced understanding of cultural subtleties and effective communication skills is pivotal for mission success and fostering positive relations [88]. Real-world training initiatives, encompassing language courses and immersive cultural exercises, have consistently demonstrated their effectiveness in augmenting soldiers’ capabilities to operate adeptly in foreign environments. By honing linguistic proficiency and cultural acumen, soldiers bolster their effectiveness in the field and contribute to fostering diplomatic and cooperative relationships, essential components in the intricate landscape of contemporary military endeavours [88]. Finally, integrating unmanned systems and robotics into training underscores the dynamic evolution of modern warfare. Immersing soldiers in practical exercises involving drones, uncrewed vehicles, and robotic systems equips them for operations entailing these cutting-edge technologies, spanning surveillance, logistics, and combat support. Hands-on training with these systems ensures soldiers develop proficiency in their utilisation during real-world scenarios, thereby amplifying operational capabilities and bolstering mission effectiveness. By familiarising military personnel with the intricacies of unmanned systems, this training approach enhances adaptability and readiness, crucial elements in navigating the complexities of contemporary military landscapes [89].

In summary, the shift from traditional drill-based training to real-world military applications signifies a profound evolution in military preparedness. Embracing advanced simulations, LVC training environments, resilience training, wearable technology, cultural training, and unmanned systems, these applications equip soldiers with a comprehensive skill set, valuable experience, and the adaptability required for the complexities of modern warfare.

## 9. Conclusions and Visions for Future Military Training

In the intricate landscape of military training, the intricacies of research and implementation exhibit significant variation across different branches of the armed forces—the Army, Navy, Air Force, and Marine Corps—owing to their unique operational demands. This variability stems from the distinct environments in which each branch operates. For instance, with its high-tech operations, the Air Force prioritises advanced flight simulators and aerial combat training technologies. In contrast, the Army focuses on ground tactics, urban warfare simulations, and physical endurance training to align with its operational requirements. Due to its maritime focus, the Navy invests in underwater and surface warfare training. At the same time, the Marine Corps, often engaged in amphibious operations, necessitates a hybrid approach combining naval and ground combat training. Therefore, this diversity in training needs and focus areas makes military training research a complex, multifaceted endeavour. It goes beyond developing the latest technology, requiring a deep understanding of each military branch’s specific requirements and constraints. Additionally, the rate of technological adoption and the availability of resources for training vary, resulting in disparities in the speed and effectiveness of implementing new training methods.

A significant challenge in military training research will be to develop adaptable and flexible training systems that cater to the diverse needs of different branches. This involves creating modular training programs customisable for specific environments and operational scenarios. There is also a growing need to balance high-tech training solutions with traditional, time-tested methods to ensure comprehensive preparation for soldiers. Another critical area for future research is integrating psychological and mental health training across all branches. While progress has been made in understanding and addressing the psychological impacts of military service, this remains an area where more uniform advancements are needed. Ensuring soldiers from all branches are equipped to handle their service’s mental and emotional challenges is crucial.

In conclusion, military training is a dynamic and complex field, marked by varying levels of advancement and focus across different branches. The future of military training research lies in developing adaptable, branch-specific methodologies while striving for advancements in areas common to all military sectors, such as mental health and resilience training. As warfare continues to evolve, so must the approaches to training, ensuring that all military personnel, regardless of their branch, are thoroughly prepared for their challenges.

## Data Availability

Data are contained within the article.
